# Long term survival of small cell lung cancer patients after chemotherapy.

**DOI:** 10.1038/bjc.1993.150

**Published:** 1993-04

**Authors:** A. van der Gaast, P. E. Postmus, J. Burghouts, C. van Bolhuis, J. Stam, T. A. Splinter

**Affiliations:** Department of Medical Oncology, University Hospital Rotterdam-Dijkzigt, The Netherlands.

## Abstract

Eighty-one patients with small cell lung cancer (SCLC) with a survival of more than 2 years after start of chemotherapy were studied. Twenty-six of the 28 patients who died of relapsed SCLC had in fact relapsed before two years and of the 55 who had not then only two (4%) relapsed subsequently. It is stressed that with such observations treatment related factors should be taken in account. Second tumours were observed in ten patients, nine proven malignant. Of the eight patients with non-small cell lung cancer three had residual disease after initial chemotherapy. In our patient group after a 2 year disease-free interval the risk of developing non-small cell lung cancer seems higher than a subsequent relapse of SCLC.


### Long term survival of small cell lung cancer patients after chemotherapy

A. van der Gaast[1], P.E. Postmus[2], J. Burghouts[3], C. van Bolhuis[1], J. Stam[4] & T.A.W. Splinter[1]

[1]*Department of Medical Oncology, University Hospital Rotterdam-Dijkzigt;* [2]*Department of Pulmonary Diseases, University Hospital Groningen;* [3]*Department of Internal Medicine, Groot Ziekengasthuis, 's-Hertogenbosch;* [4]*Department of Pulmonary Diseases, Free University Hospital, Amsterdam, The Netherlands.*

**Summary** Eighty-one patients with small cell lung cancer (SCLC) with a survival of more than 2 years after start of chemotherapy were studied. Twenty-six of the 28 patients who died of relapsed SCLC had in fact relapsed before two years and of the 55 who had not then only two (4%) relapsed subsequently. It is stressed that with such observations treatment related factors should be taken in account. Second tumours were observed in ten patients, nine proven malignant. Of the eight patients with non-small cell lung cancer three had residual disease after initial chemotherapy. In our patient group after a 2 year disease-free interval the risk of developing non-small cell lung cancer seems higher than a subsequent relapse of SCLC.

In recent years a number of studies have been published concerning prognostic factors of patients with small-cell lung cancer (SCLC). Most patients who achieve long-term survival after treatment for SCLC initially present with favourable prognostic factors such as good performance status, limited disease, female gender, few biochemical abnormalities and good response to treatment (Albain *et al.*, 1990; Osterlind & Anderson, 1986; Spiegelman *et al.*, 1989). However when these patients have reached a survival of more than 2 or 3 years after start of treatment such factors have lost their predictive value for further disease-free survival or subsequent relapse.

A number of studies have focused solely on those patients who achieve long-term survival (Davis *et al.*, 1985; Niiranen, 1988; Jackson *et al.*, 1988; Pallares *et al.*, 1987; Souhami & Law, 1990). In order to gather more knowledge about the fate of patients with SCLC who obtain long-term survival we performed a retrospective study of all consecutively treated patients in four different centres with a survival of more than 24 months after start of treatment. Special attention was paid to subsequent relapse and the development of secondary malignancies. No attempt was made to study the differences in pretreatment prognostic factors between this group of patients and the parent group since these factors are well known from the literature.

### Patients and methods

The medical records of all patients with SCLC defined according to the morphological criteria of the WHO (1982) and diagnosed between 1980 and 1989 in four different centres were reviewed. Eighty-one patients were identified who survived more than 2 years after start of chemotherapy. Minimal staging procedures for almost all patients had included physical examination, chest X-ray, computed tomography scan or ultrasound of the upper abdomen, bone scan and unilateral bone-marrow biopsy. Limited disease (LD) was defined as disease confined to one hemithorax including ipsilateral hilar, mediastinal lymph nodes, and supraclavicular lymph nodes and extensive disease (ED) as disease spread beyond the hemithorax including extension to the chest wall or to the contralateral lung. In 52 patients the initial chemotherapy regimen consisted of doxorubicin, cyclocphosphamide, and etoposide. Ten patients were treated with the combination of cisplatin, cyclophosphamide and etoposide. Eight patients were treated with a combination consisting of cyclphosphamide, vincristine and procarbazine in four patients combined with CCNU. Four patients were treated with the combination of cisplatin, doxorubicin and etoposide and three with a combination of cyclophosphamide, vincristine and etoposide. Two patients were treated with carboplatin and ifosfamide and one patient with carboplatin and vincristine. Another patient received a combination of cyclophosphamide, methotrexate and CCNU. Nine of these patients received chemotherapy after surgical resection of the tumour.

Tumour response was evaluated by standard WHO criteria (1979). Survival was recorded from the start of treatment to death or last follow-up. Survival curves were calculated according to the method of Kaplan-Meier. Additional clinical characteristics are shown in Table I.

### Results

The overall survival curve of the patients from 2 years after start of chemotherapy is shown in Figure 1. At the time of analysis 43 patients had died with a median survival of 143 weeks (range 106-464). The causes of death are listed in Table II. The causes of death were available from the hospital records for most patients. In some instances further information was obtained from the patients general physician to obtain additional information about the cause of death.

Twenty-eight patients died from progressive SCLC. Twenty-one, five and two patients died in the third, fourth and fifth year respectively. The survival curve in Figure 2 shows the death from SCLC censored for non-SCLC deaths. Twenty-six of these 28 patients had already evidence of disease progression before they had reached a survival 2 years after start of treatment. More than 60% of the twenty-three patients treated with reinduction chemotherapy achieved an objective response to second-line treatment. The two patients who had no evidence of disease 24 months after

**Table I** Patient characteristics

| | |
|---|---|
| No. patients | 81 |
| Median age (years) | 60 (range 35-74) |
| Sex | |
| male | 65 (80%) |
| female | 16 (20%) |
| Median performance status (Karnofsky) | 90% (range 30-100) |
| Limited disease | 68 (84%) |
| Extensive disease | 13 (16%) |
| Response | |
| complete response | 65 (80%) |
| partial response | 6 (7%) |
| stable disease | 1 (1%) |
| non-evaluable (adjuvant chemotherapy) | 9 (11%) |
| Chest irradiation | 33 (41%) |
| Prophylactic cranial irradiation | 60 (74%) |

Correspondence: A. van der Gaast, Department of Medical Oncology, University Hospital Rotterdam-Dijkzigt, Dr. Molewaterplein 40, 3015 GD Rotterdam, The Netherlands.
Received 4 February 1992; and in revised form 11 November 1992.

**Figure 2** Survival curve of 81 patients with small cell lung cancer with a survival of more than 2 years after start of treatment.

**Table II** Causes of death

| | | No. of patients |
|---|---|---|
| Recurrent SCLC | | 28 |
| Second malignancies | | 9 |
| Non small cell lung cancer | 8 | |
| Non-Hodgkin lymphoma | 1 | |
| Recurrent pulmonary tumour (no histology) | | 1 |
| Non-malignant causes | | 3 |
| Unknown | | 2 |

start of chemotherapy relapsed 30 and 50 months after start of treatment, one with liver and bone metastases and the other with brain metastases.

Second tumours were observed in ten patients, nine proven malignant. Of the eight patients with non-small cell lung cancer three had residual disease after initial chemotherapy which proved tobe non-small lung cancer (one adenocarcinoma and two squamous cell carcinoma). It seems probable that these three patients had tumours with a mixed histology at presentation. The other five patients developed non-small cell lung cancer 19, 47, 56, 60 and 84 months after diagnosis of SCLC. All these five patients had a histological diagnosis of squamous cell carcinoma. Although five of these patients were operated and three were treated with radiotherapy all eight patients died due to distant metastases of non-small cell lung cancer. One patient developed and died due to a non-Hodgkin lymphoma of the small intestines at 7 years. The chemotherapy regimen of this patients had included a combination of cyclophosphamide, vincristine and procarbazine which might have attributed to the development of this non-Hodgkin lymphoma. One patient died due to a recurrent pulmonary tumour 7 years after diagnosis of SCLC but no histological diagnosis could be obtained.

**Figure 2** Death from small cell lung cancer alone, death from non-small cell lung cancer censored.

Non-malignant causes of death were found in three patients. In two patients the cause of death was unknown. Of the nine patients who received chemotherapy after surgical resection of the tumour, five patients are still alive without recurrent disease and a median survival of 70 months (range 52–120).

Of the 13 patients with extensive disease all patients had metastases to only one organ site. Four of these patients have still no evidence of disease with a follow-up of 65, 69, 72 and 80 months after start of chemotherapy. There was not a significant difference between the survival curves of the patients who were initially staged as limited or extensive disease beyond a survival of more than 2 years.

### Discussion

Although SCLC is a chemosensitive disease the number of patients surviving for more than 2 years is still low and relapses as late as 8 years after diagnosis have been reported (Vogelsang *et al.*, 1985). As can be seen in Table I most of the patients who achieved long-term survival presented with initial favourable prognostic factors and most patients had few biochemical abnormalities.

Of the 81 patients with a survival of more than 24 months after start of treatment 35% died from progressive SCLC up till 55 months. The overall survival curve is quite similar to those reported by Souhami & Law (1990). Remarkably only two of the 55 patients (4%) with a disease-free survival of more than 2 years have relapsed after a median follow-up of 5.7 years. Osterlind *et al.* (1986) reported on 72 patients disease-free at restaging 18 months after start of treatment. During the first 6 months after restaging 19 patients relapsed and 14 of 53 patients (26%) relapsed after a disease-free interval of more than 24 months with a minimum follow-up of 4 years. One explanation for this observed difference in relapse rates may be that in contrast to the Danish study 75% of our patients did not receive chemotherapy for a duration of more than 6 months. Although maintenance treatment has probably no impact on overall survival it seems to increase the disease-free interval (Spiro *et al.*, 1989; Splinter, 1988). Therefore patients who have received chemotherapy for 18 months and who are still disease-free at two years have a higher relapse rate than patients who have received chemotherapy for a shorter period. The favourable survival of some of the patients who had already relapsed before they had reached a survival for more than 2 years and the high response to reinduction chemotherapy also argues for this hypothesis (Postmus *et al.*, 1987).

Lung tumours with a non-small cell histology were encountered in eight patients. In three patients the non-small cell histology became apparent in the residual tumour after they had been given treatment for SCLC. Five patients were diagnosed with non-small cell carcinoma of the lung 19–84 months after the diagnosis of SCLC. Johnson *et al.* (1986) estimated the risk of development of non-small cell lung carcinoma after two years of disease-free survival 4.4% per person year. Since in our study 55 patients were disease-free at 24 months with a median follow-up of 5.7 years the expected number of patients who had developed non-small cell lung cancer would have been 8 patients. Despite the fact that all of these patients had been followed on a regular basis and therefore theoretically these tumours should have been detected at a rather early stage and furthermore at least five patients had operable disease at presentation, all these patients died due to distant metastases. Data about the prognosis of patients who develop a second primary lung carcinoma after small cell carcinoma are scarce. Craig *et al.* (1984) reported on ten patients of whom the survival after diagnosis of the second primary was greater than 6 months in only one patient. In the earlier mentioned study of Johnson no patient survived more than 40 weeks from histologic documentation of the second cancer (Johnson *et al.*, 1986). Until now there is no obvious explanation for the dismal prognosis of these patients.

In conclusion we observed in this study a low relapse rate (4%) of SCLC after a disease-free interval of more than 2 years. It is stressed that with such observations treatment related factors should be taken in account. Since after a 2 year disease-free interval the risk of developing NSCLC seems higher than a subsequent relapse of SCLC follow-up should be focused on early detection of a second primary. More data are required to show whether the prognosis of those patients who develop a second primary NSCLC is worse than those patients who develop a second primary NSCLC than of patients who develop NSCLC without antecedent SCLC and whether or not chemo-prevention trials in such a high risk group may be worthwhile.

### References

ALBAIN, K.S., CROWLEY, J.J., LEBLANC, M. & LIVINGSTON, R.B. (1990). Determinants of improved outcome in small-cell lung cancer: an analysis of the 2,580-patient Southwest Oncology Group data base. *J. Clin. Oncol.*, **8**, 1563–1574.

CRAIG, J., POWELL, B., MUSS, H.B., KAWAMOTO, E. & BREYER, R. (1984). Second primary bronchogenic carcinomas after small cell carcinomas. *Am. J. Med.*, **76**, 1013–1020.

DAVIS, S., WRIGHT, P.W., SCHULMAN, S.F., SCHOLES, D., THORNING, D. & HAMMAR, S. (1985). Long-term survival in small-cell carcinoma of the lung: a population experience. *J. Clin. Oncol.*, **3**, 80–91.

JACKSON, D.V., CASE, L.D., ZEKAN, P.J., POWELL, B.L., CALDWELL, R.D., BEARDEN, J.D., NELSON, E.C., MUSS, H.B., COOPER, R., RICHARDS, F., WHITE, D.R., CRUZ, J.M., CAPONERA, M.E., FURR, C.S., SPURR, C.L. & CAPIZZI, R.L. (1988). Improvement of long-term survival in extensive small-cell lung cancer. *J. Clin. Oncol.*, **6**, 1161–1169.

JOHNSON, B.E., IHDE, D.C., MATTHEWS, M.J., BUNN, P.A., ZABELL, A., MAKUCH, R.W., JOHNSTON-EARLY, A., COHEN, M.H., GLATSTEIN, E. & MINNA, J.D. (1986). Non-small-cell lung cancer. Major cause of late mortality in patients with small cell lung cancer. *Am. J. Med.*, **80**, 1103–1110.

NIIRANEN, A. (1988). Long-term survival in small cell carcinoma of the lung. *Eur. J. Cancer Clin. Oncol.*, **24**, 749–752.

OSTERLIND, K. & ANDERSON, P.K. (1986). Prognostic factors in small cell lung cancer: a multivariate model based on 778 patients treated with chemotherapy with or without irradiation. *Cancer Res.*, **46**, 4189–4194.

OSTERLIND, K., HANSSEN, H.H., HANSEN, M., DOMBERNOWSKY, P. & ANDERSEN, P.K. (1986). Long-term disease-free survival in small-cell carcinoma of the lung: a study of clinical determinants. *J. Clin. Oncol.*, **4**, 1307–1313.

PALLARES, C., BASTUS, R., LOPEZ, J.J. & DE ANDRES, L. (1987). Long-term survival in small cell carcinoma of the lung. *Eur. J. Cancer Clin. Oncol.*, **23**, 541–544.

POSTMUS, P.E., BERENDSEN, H.H., VAN ZANDWIJK, N., SPLINTER, T.A.W., BURGHOUTS, J.T.M., BAKKER, W. & THE EORTC LUNG CANCER COOPERATIVE GROUP (1987). Retreatment with the induction regimen in small cell lung cancer relapsing after an initial response to short term chemotherapy. *Eur. J. Cancer Clin. Oncol.*, **23**, 1409–1411.

SOUHAMI, R.L. & LAW, K. (1990). Longevity in small cell lung cancer. A report to the lung cancer subcommittee of the United Kingdom coordinating Committee for cancer research. *Br. J. Cancer*, **61**, 584–589.

SPIEGELMAN, D., MAURER, L.H., WARE, J.H., PERRY, M.C., CHAHINIAN, A.P., COMIS, R., EATON, W., ZIMMER, B. & GREEN, M. (1989). Prognostic factors in small-cell carcinoma of the lung: an analysis of 1,521 patients. *J. Clin. Oncol.*, **7**, 344–354.

SPIRO, S.G., SOUHAMI, R.L. & GEDDES, D.M. (1989). Duration of chemotherapy in small cell lung cancer: a Cancer Research Campaign trial. *Br. J. Cancer*, **59**, 578–583.

SPLINTER, T.A.W. FOR THE EORTC LUNG CANCER COOPERATIVE GROUP (1988). EORTC 08825: induction versus induction plus maintenance chemotherapy in small cell lung cancer. Definitive evaluation. *Proc. ASCO*, **7**, 202.

VOGELSANG, G.B., ABELOFF, M.D., ETTINGER, D.S. & BOOKER, S.V. (1985). Long-term survivors of small cell carcinoma of the lung. *Am. J. Med.*, **79**, 49–56.

WHO (1979). Handbook of Reporting Results of Cancer Treatment. Geneva. World Health Organization. Offset publication no. 48.

WHO(1982). The World Health Organization histological typing of lung tumours. *Am. J. Clin. Pathol.*, **74**, 123–136.


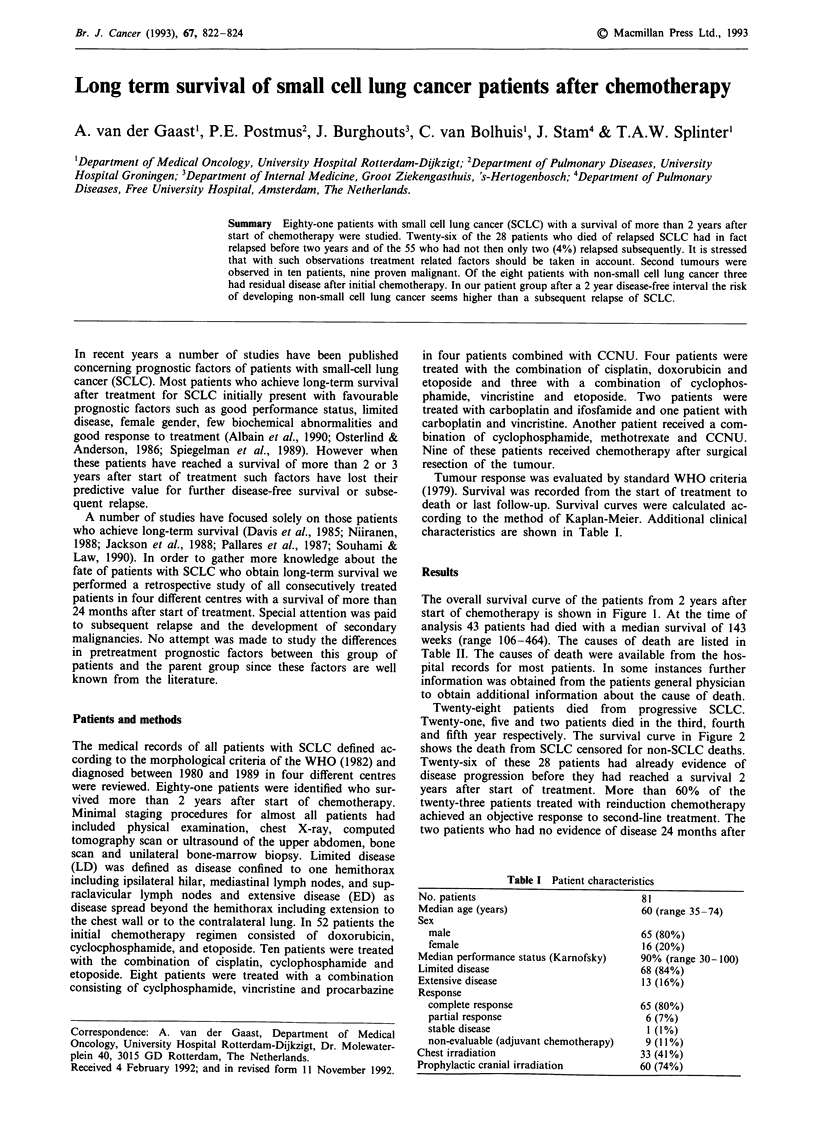

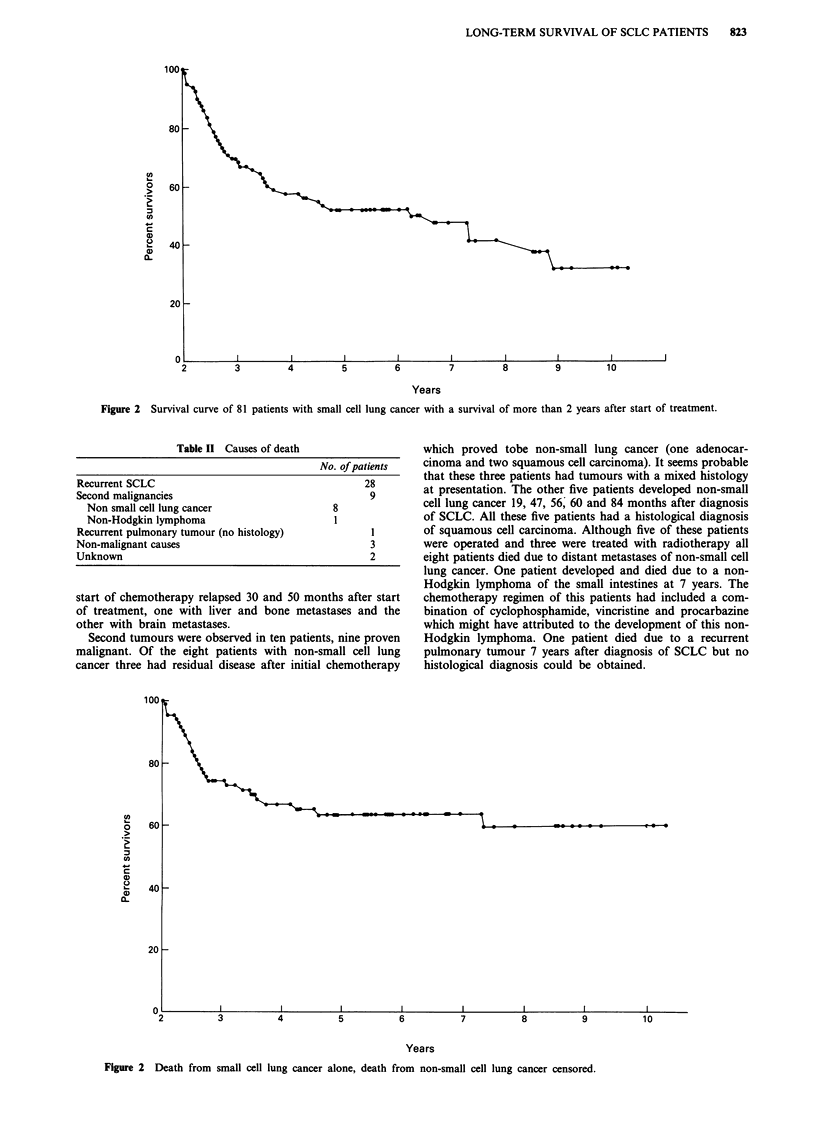

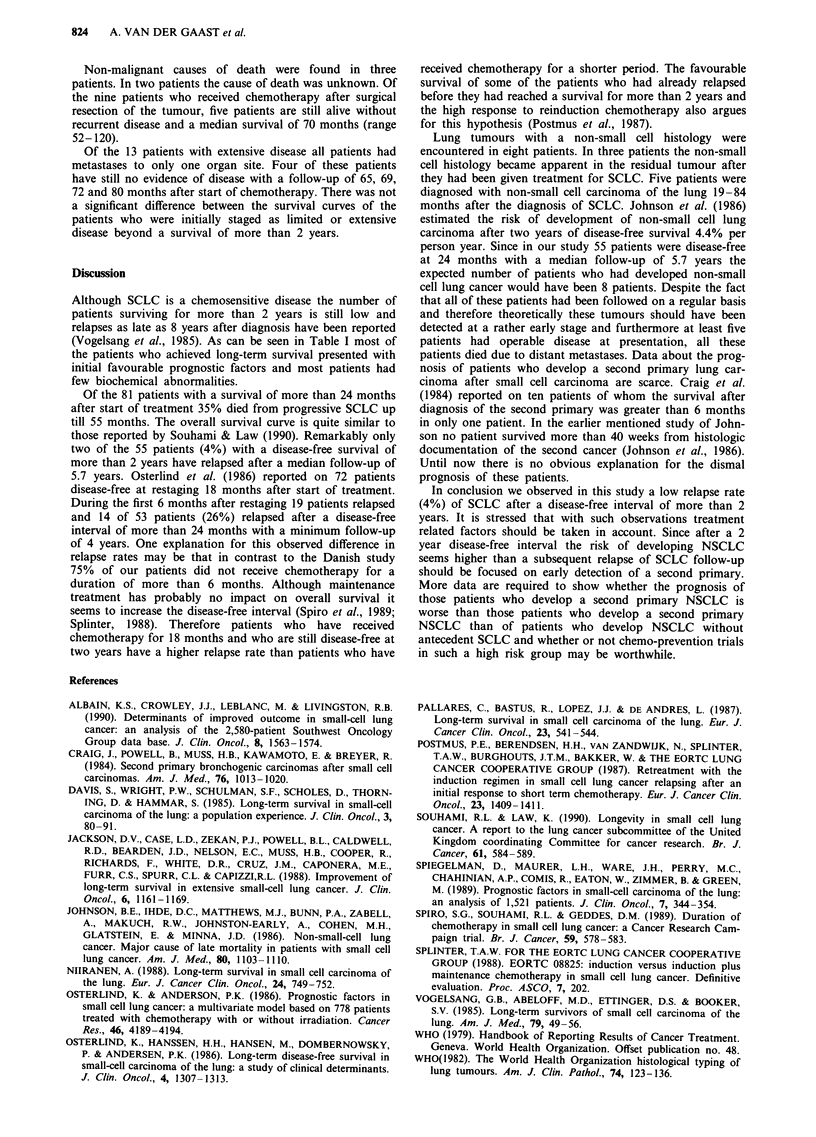


## References

[OCR_00330] Albain K. S., Crowley J. J., LeBlanc M., Livingston R. B. (1990). Determinants of improved outcome in small-cell lung cancer: an analysis of the 2,580-patient Southwest Oncology Group data base.. J Clin Oncol.

[OCR_00336] Craig J., Powell B., Muss H. B., Kawamoto E., Breyer R. (1984). Second primary bronchogenic carcinomas after small cell carcinoma. Report of two cases and review of the literature.. Am J Med.

[OCR_00343] Davis S., Wright P. W., Schulman S. F., Scholes D., Thorning D., Hammar S. (1985). Long-term survival in small-cell carcinoma of the lung: a population experience.. J Clin Oncol.

[OCR_00347] Jackson D. V., Case L. D., Zekan P. J., Powell B. L., Caldwell R. D., Bearden J. D., Nelson E. C., Muss H. B., Cooper M. R., Richards F. (1988). Improvement of long-term survival in extensive small-cell lung cancer.. J Clin Oncol.

[OCR_00355] Johnson B. E., Ihde D. C., Matthews M. J., Bunn P. A., Zabell A., Makuch R. W., Johnston-Early A., Cohen M. H., Glatstein E., Minna J. D. (1986). Non-small-cell lung cancer. Major cause of late mortality in patients with small cell lung cancer.. Am J Med.

[OCR_00362] Niiranen A. (1988). Long-term survival in small cell carcinoma of the lung.. Eur J Cancer Clin Oncol.

[OCR_00366] Osterlind K., Andersen P. K. (1986). Prognostic factors in small cell lung cancer: multivariate model based on 778 patients treated with chemotherapy with or without irradiation.. Cancer Res.

[OCR_00372] Osterlind K., Hansen H. H., Hansen M., Dombernowsky P., Andersen P. K. (1986). Long-term disease-free survival in small-cell carcinoma of the lung: a study of clinical determinants.. J Clin Oncol.

[OCR_00378] Pallares C., Bastus R., Lopez J. J., De Andres L. (1987). Long-term survival in small cell carcinoma of the lung.. Eur J Cancer Clin Oncol.

[OCR_00383] Postmus P. E., Berendsen H. H., van Zandwijk N., Splinter T. A., Burghouts J. T., Bakker W. (1987). Retreatment with the induction regimen in small cell lung cancer relapsing after an initial response to short term chemotherapy.. Eur J Cancer Clin Oncol.

[OCR_00391] Souhami R. L., Law K. (1990). Longevity in small cell lung cancer. A report to the Lung Cancer Subcommittee of the United Kingdom Coordinating Committee for Cancer Research.. Br J Cancer.

[OCR_00397] Spiegelman D., Maurer L. H., Ware J. H., Perry M. C., Chahinian A. P., Comis R., Eaton W., Zimmer B., Green M. (1989). Prognostic factors in small-cell carcinoma of the lung: an analysis of 1,521 patients.. J Clin Oncol.

[OCR_00403] Spiro S. G., Souhami R. L., Geddes D. M., Ash C. M., Quinn H., Harper P. G., Tobias J. S., Partridge M., Eraut D. (1989). Duration of chemotherapy in small cell lung cancer: a Cancer Research Campaign trial.. Br J Cancer.

[OCR_00414] Vogelsang G. B., Abeloff M. D., Ettinger D. S., Booker S. V. (1985). Long-term survivors of small cell carcinoma of the lung.. Am J Med.

